# LC–MS/MS method for the quantitation of a dual PI3K/BRD4 inhibitor SF2523 in mouse plasma: Application to plasma protein binding and metabolism studies

**DOI:** 10.1002/bmc.5643

**Published:** 2023-04-27

**Authors:** Veenu Bala, Yashpal S. Chhonker, Guillermo A. Morales, Krishnaiah Maddeboina, Dhananjaya Pal, Donald L. Durden, Daryl J. Murry

**Affiliations:** 1 Clinical Pharmacology Laboratory, Department of Pharmacy Practice and Science, University of Nebraska Medical Center, Omaha, Nebraska, USA; 2 SignalRx Pharmaceuticals, Inc., Cumming, Georgia, USA; 3 Molecular Targeted Therapeutics Laboratory, Atrium Health-Levine Cancer Institute, Charlotte, North Carolina, USA; 4 Atrium Health Wake Forest Baptist Comprehensive Cancer Center, Winston Salem, North Carolina, USA; 5 Division of Pediatric Hematology–Oncology, Department of Pediatrics, Moores Cancer Center, UC San Diego School of Medicine, La Jolla, California, USA; 6 Fred and Pamela Buffett Cancer Center, University of Nebraska Medical Center, Omaha, Nebraska, USA

**Keywords:** dual PI3K/BRD4 inhibitor, LC–MS/MS, SF2523

## Abstract

A sensitive and selective liquid chromatography coupled with tandem mass spectrometry (LC–MS/MS) method was developed and validated for the quantitation of dual PI3K/BRD4 inhibitor SF2523 in mouse plasma. The analysis was performed on a UPLC system connected to a Shimadzu 8060 mass spectrometer by electrospray ionization in positive multiple reaction monitoring mode. Chromatographic separation was carried out on an ACE Excel C_18_ column with a gradient elution containing 0.1% formic acid and methanol as the mobile phase. The linearity was conducted in the concentration range 0.1-500 ng/ml for SF2523 in 100 μl of plasma. The inter- and intra-batch precision (RSD) were both lower than 13.5%, with the accuracy (percentage bias) ranging from −10.03 to 11.56%. The validated method was successfully applied to plasma protein binding and in vitro metabolism studies. SF2523 was highly bound to mouse plasma proteins (>95% bound). Utilizing mouse S9 fractions, a total of seven phase I and II metabolites were identified with hydroxylation found to be the major metabolic pathway. Metabolite identification included analysis of retention behaviors, molecular weight changes and MS/MS fragment patterns of SF2523 and the metabolites. This newly developed and validated method allows the rapid and easy determination of the SF2523 concentration with high sensitivity in a low sample volume and can be applied to future pre-clinical studies.

## 1 ∣ INTRODUCTION

SF2523 is a thienopyranone (TP) scaffold small molecule that contains a 2,3-dihydrobenzo[*b*][1,4]dioxin moiety on the 3-position of the thiophene ring and a morpholino group on the 5-position of the pyranone ring system (Morales et al., 2013). SF2523 is a structure-based designed second-generation potent phosphatidylinositol 3-kinase (PI3K) small-molecule inhibitor with an impressive augmented drug profile over first-generation PI3K inhibitor LY294002 (Vlahos et al., 1994). Structure–activity relationship studies revealed that the phenyl and morpholino functional groups on the TP ring system, as well as the carbonyl unit of the pyranone, are critical for PI3K enzyme affinity and inhibition (Morales et al., 2013). SF2523 is reported to be a highly potent dual PI3K and BRD4 inhibitor that blocks tumor growth and metastasis in various cancer models (Andrews et al., 2017; Carlino & Rastelli, 2016; Joshi et al., 2019; Morales et al., 2013).

Phosphoinositide 3-kinases are lipid kinase enzymes that transduce signals arising from cell surface receptors such as tyrosine kinase receptors and G-protein coupled receptor to the inside of the nucleus. PI3K mechanistically targets rapamycin and signaling pathways that play a pivotal role in a variety of cellular processes, including cell proliferation, differentiation, survival, metabolism and angiogenesis (Janku et al., 2018; Laplante & Sabatini, 2012; Saxton & Sabatini, 2017; Shimobayashi & Hall, 2014; Thorpe et al., 2015; Wymann & Schneiter, 2008). Bromodomain and extra terminal domain (BET) containing proteins are important for recognizing acetylated lysines on chromatin (Gallenkamp et al., 2014). The targeting of BET proteins is a research area of increasing interest for the treatment of cancer, inflammation and viral infectious diseases (Bandukwala et al., 2012; Boehm et al., 2013; Delmore et al., 2011; Filippakopoulos & Knapp, 2014). BRD4 binds directly to acetylated histones through the process of mitosis, which is essential for the retention of a chromatin structure in the daughter cells (Vidler et al., 2012).

A wide range of BRD4 as well as pan BET inhibitors have emerged that are currently in preclinical developmental stages with some of them already undergoing clinical development. However, an urgent need for more potent and efficacious BET inhibitors remains because mechanistic studies have shown that BET inhibitors lose their efficacy owing to drug resistance buildup in cancer cells, as demonstrated by the activation of compensatory survival signaling pathways to circumvent the BET inhibitors’ drug action (Dai et al., 2017; Rathert et al., 2015; Zhang et al., 2017). This crippling limitation can be overcome by targeting multiple pathways simultaneously that are known to support cancer cell growth and survival. This could be achieved through a combinatorial treatment approach using two individual single-target drugs or a multi-target single-drug molecule. The latter strategy is strongly supported by recent studies demonstrating that TP-based multi-action BET inhibitors are more effective than single-molecule chemotherapeutic agents against a variety of cancers (Vann et al., 2020). SF2523 orthogonally inhibits PI3K and BRD4 simultaneously to block the expression and activation of oncoprotein MYC and, therefore, reduces tumor burden and metastasis in different preclinical cancer models (Andrews et al., 2017). Furthermore, in addition to its antitumor activity, SF2523 plays a pivotal role in inducing an antitumor immune response via macrophage polarization (Joshi et al., 2019), and SF2523 also suppresses human immunodeficiency virus type-1 replication in macrophage through autophagy (Campbell et al., 2018).

To elucidate the concentration–effect relation and potential off-target concerns of this dual PI3K/BRD4 inhibitor, a sensitive, reproducible and precise analytical method is required. Although several preclinical studies of SF2523 have been reported, no absorption, distribution, metabolism and elimination (ADME) studies or in-depth analytical or bioanalytical method of SF2523 have been reported. Because the LC–MS/MS method is widely used for the determination of small molecules in a biological matrix, we established and validated a simple and sensitive UHPLC–MS/MS method for the determination of SF2523 in mouse plasma. The rapid, reliable and targeted bio-analysis of drug and metabolite concentrations in plasma samples is essential for pharmacokinetic, pharmacodynamic and toxicokinetic studies. Herein, we describe the first high-throughput, sensitive and specific method for determining plasma concentrations of SF2523 in murine models. Our goal was to develop an LC–MS/MS method for the quantitation of SF2523 in mouse plasma with a low limit of detection of 0.1 ng/ml using 100 μl of plasma sample. In addition, we assessed gastrointestinal stability, blood-to-plasma ratio, plasma protein binding, and the in vitro metabolism and metabolite identification of SF2523. Metabolite identification provides us a quick look at the metabolic fate of SF2523 and resolves the major metabolic pathways. This method is flexible and expandable according to study needs and enables the retrospective data analysis to identify the novel metabolites.

## 2 ∣ MATERIALS AND METHODS

### 2.1 ∣ Chemicals and materials

SF2523 (purity ≥98%) and SRX-3334 (purity ≥98%) were kindly provided by SignalRx Pharmaceuticals Inc., with SRX-3334 utilized as an internal standard (IS). LC–MS-grade methanol and acetonitrile were purchased from Fisher Scientific. Bond Elute solid-phase extraction cartridges (C_18_, 50 mg per 1 ml) were purchased from Agilent Technologies Inc. (Agilent Technologies, Inc., Santa Clara, CA, USA). Ultrapure water was generated using a GenPUre xCAD plus water purification system (Thermo Fisher Scientific). Mouse plasma was purchased from Equitech-Bio Inc. (Kerrville, TX, USA). Mouse liver S9 fractions and microsomes were purchased from XenoTech LLC. (Lenexa, KS, USA). All other materials and reagents were purchased from standard chemical suppliers and of analytical grade or higher.

### 2.2 ∣ Liquid chromatography and mass spectrometry conditions

The LC–MS/MS system was composed of a Shimadzu 8060 mass and Nexera Series (Shimadzu Scientific Instruments, Columbia, MD, USA) liquid chromatograph. The analytical column was an ACE Excel C_18_ (1.7 μm, 100 × 2.1 mm, Advance Chromatography Technologies Ltd, UK) equipped with a Phenomenex C_18_ guard column (Phenomenex, Torrance, CA, USA), which was used to separate the analytes and the internal standard. The mobile phase consisted of water containing 0.1% formic acid (phase A) and methanol (phase B) at a flow rate of 0.25 ml/min with an operating temperature of room temperature. The entire elution system was set to last 6.0 min with the following gradient elution system: 35% B, increasing to 90% B over 2.0 min, then held constant for 2.5 min, and finally brought back to the initial condition of 35% B in 0.20 min followed by 1 min of re-equilibration. The injection volume (2 μl) was consistent for all samples. The autosampler chamber was maintained at 4°C throughout the analysis.

The MS/MS system was operated at unit resolution in multiple reaction monitoring (MRM) mode in positive electrospray ionization mode. The mass spectrometer source settings were optimized to the following: nebulizer gas, 2.0 L/min; heating gas, 10 L/min; drying gas, 10 L/min; interface temperature, 375°C; desolvation line temperature, 220°C; and heat block temperature, 300°C. The MRM transitions for SF2523 and IS, as well as their respective optimum MS parameters, such as voltage potential (Q1, Q3) and collision energy (CE), are shown in Table 1. Data acquisition and quantitation were performed using LabSolutions software version 5.80 (Shimadzu Scientific Inc, Columbia, MD, USA).

### 2.3 ∣ Preparation of stock, calibration standards and quality control samples

A standard independent stock solution of SF2523 and IS was prepared in duplicates at 1 mg/ml in DMSO and methanol, respectively. The following working calibration stocks (CS) were prepared by diluting stock solutions with methanol: 5.0, 4.0, 0.5, 0.1, 0.01, 0.005, 0.002 and 0.001 μg/ml. All stock solutions and working stocks were stored at −20°C until use. Calibration curve (CC) samples were prepared in mouse plasma by spiking 90 μl plasma with 10 μl CSs: 500, 400, 50, 10, 1, 0.5, 0.2 and 0.1 ng/ml. The quality controls (QCs) were prepared in the same way to obtain the desired concentrations at: 0.1 ng/ml, the lower limit of quantification (LLOQ); 0.3 ng/ml, the low quality control (LQC); 200 ng/ml, the medium quality control (MQC); and 375 ng/ml, the high quality control (HQC). All CCs and QCs were prepared fresh as needed.

### 2.4 ∣ Plasma and liver S9 fraction sample preparation

The CC, QCs and plasma samples were extracted using Agilent Bond Elute C_18_, 50 mg per 1 ml solid phase extraction (SPE) cartridges (Agilent Technologies, Inc., Santa Clara, CA, USA). Briefly, CS (10 μl) or QC (10 μl) and IS (10 μl from 0.5 μg/ml stock) were added to 90 μlof mouse plasma. Next, 600 μl of 1% formic acid (FA) in water was added, and the samples were vortexed. Next, 700 μl of the above prepared mixed solution was loaded on an SPE cartridge pre-conditioned with 1 ml methanol first and then 1 ml water. Then SPE column loaded with the sample was washed with 1 ml of 15% methanol. Subsequently, analytes were eluted with two aliquots of methanol (0.5 ml). Finally, the SPE eluent was dried at 50°C under nitrogen, reconstituted using 100 μl of 0.1% FA–methanol (60:40) and centrifuged (17,950**g**, 4°C) for 10 min. The resultant supernatant was transferred into autosampler vials (~80 μl) and 2 μl of the sample was injected into LC–MS/MS.

For metabolic stability samples, extractions (50 μl) were taken at each pre-determined time point and mixed with 300 μl of ice-cold methanol containing IS in a 1.5 ml centrifuge tube. Then, samples were vortexed for 2 min and centrifuged (17,950**g**) for 10 min. An aliquot (100 μl) of the clear supernatant was transferred into an autosampler vial and 2 μl was injected into the LC–MS/MS system.

### 2.5 ∣ Assay validation

The LC–MS/MS method was developed and validated in plasma and partially validated in microsomes and the S9 fraction, per the current guidelines of the US Food and Drug Administration (FDA, 2018).

Interference from endogenous compounds was investigated by analysis of three different blank plasma samples. Potential interference by endogenous compounds at the retention time of the analytes or IS was evaluated. Assay sensitivity was quantified using the signal-to-noise ratio (S/N) of each analyte in the CSs, and the LOD and LLOQ were defined as peak areas with S/N ratios of 3 and 10, respectively.

The linearity of the method was assessed by analyzing three complete standard curves on three separate days. The CC was processed by peak area, relative to one of the IS samples. The mean value and coefficient of variation (CV) for the slopes, intercepts and correlation coefficients obtained from the three calibration curves were calculated. Each CS and QC was required to be ±15% standard deviation (SD) of the expected value except for the LLOQ, which was required to be ±20%. CSs were run in ascending order followed by two consecutive “zero samples” to assess any carryover, which was required to be <20% of the expected S/N for LLOQ.

The inter-day and intra-day accuracy and precision were determined by analyzing variations in the QCs calculated concentration. This was performed in five replicates over three consecutive days, wherein samples were prepared in mouse plasma as previously described. Precision was defined as the percentage relative standard deviation (RSD) and accuracy was defined as the percentage bias (bias) with acceptance criteria of ±15% (except for ±20% at LLOQ) for both.


(1) $$\%\;\text{Bias}=\frac{\left(\text{observed concentration}-\text{nominal concentration}\right)}{\text{nominal concentration}}\times 100$$

### 2.6 ∣ Recovery and matrix effect

The extraction recovery was calculated for both SF2523 and IS by comparing their respective peak areas between extracted plasma with corresponding post-extracted plasma standards at three QC concentration levels (LQC, MQC and HQC). The recovery was acceptable if consistent and reproducible.

The matrix effect (ME) was investigated on two batches of blank plasma. The ME of the mouse plasma constituents over the ionization of SF2523 was calculated using the peak areas from blank extracts spiked with analytes at three concentrations (LQC, MQC and HQC) (post-extraction addition samples), which were compared with peak areas from standard solutions spiked with analytes at the same concentration. The ME and IS-normalized ME were calculated as described in Equations (2) and (3). The variability in ME was deemed acceptable if values were within ±15 (Matuszewski et al., 1998).


(2) $$\text{ME}=\frac{\text{Mean peak area of analyte spiked post}-\text{extraction}}{\text{Mean peak area of analyte in solvent}}\times 100$$

(3) $$\text{IS}-\text{normalized ME}=\frac{\text{Mean peak area ratio of analyte}∕\text{IS spiked post}-\text{extraction}}{\text{Mean peak area ratio of analyte}∕\text{IS in solvent}}\;\;\times 100$$

### 2.7 ∣ Plasma stability studies

Stability in mouse plasma was assessed in triplicate at LQC and HQC concentrations at room temperature (6 h, 25 ± 5°C), and after three freeze–thaw cycles (−80°C) and long-term storage (60 days at −80°C). Additionally, the 24 h autosampler stability of extracted samples (at 4°C) was determined.

### 2.8 ∣ *In vitro* studies

#### 2.8.1 ∣ Gastrointestinal fluid stability studies of SF2523

The gastrointestinal fluid (GI) stability studies of SF2523 were determined in simulated GIs at a concentrations of 10 μm as described in our previous report (Aldhafiri et al., 2020; Bala et al., 2020). Simulated GIs were prepared according to United States Pharmacopoeia (USP) specifications. Briefly, SF2523 at 10 μm concentration was incubated with simulated gastric fluid (SGF). Simulated intestinal fluid (SIF) and phosphate buffer (100 mm, pH 7.4) were kept at 37°C in a shaking water bath, each in triplicate. Samples (100 μl) were collected at 0, 15, 30, 60 and 120 min. Immediately after sample collection, 5 vols of methanol containing IS were added and samples were prepared for analysis. Stability was determined as the percentage of parent compound detected at each time point relative to the concentration at 0 min in buffer (100%) (Wang et al., 2015).

#### 2.8.2 ∣ The blood-to-plasma ratio

The mouse blood–plasma partitioning ratio (B/P) of SF2523 was determined at 0.1 and 1 μg/ml concentrations as described in our previous report (Aldhafiri et al., 2020; Bala et al., 2020). At different time points (0, 30 and 60 min) aliquots (50 μl) were collected for blood analysis, and an additional sample (120 μl) was transferred to a microcentrifuge tube and centrifuged at 4,000**g** for 10 min at 4°C to separate 50 μl plasma for analysis. The whole blood and plasma samples were further processed by SPE as described above. The analyte peak area ratios were used to calculate the blood-to-plasma (B/P) ratio using Equation (4).


(4) ∕PBration=Analyte concentration in whole bloodAnalyte concentration in plasma

#### 2.8.3 ∣ Plasma protein binding study

A rapid equilibrium dialysis device system (Thermo Scientific, Rockford, IL, USA) was used to evaluate plasma protein binding (PPB) by adding a buffer to the buffer chamber and dosing solution to the sample chamber as described in our previous report (Aldhafiri et al., 2020; Bala et al., 2020). The sample contained mouse plasma spiked with 1 or 10 μm SF2523 and was added to the sample chamber. The rapid equilibrium dialysis kit top was then sealed and incubated at 37°C on an orbital shaker at 100 rpm for 5 h.

At pre-determined times, aliquots (40 μl) were removed from the sample, and buffer chambers and mixed with an equal volume of buffer or blank plasma, respectively. The samples were further processed by SPE as described above and analyzed by LC–MS/MS. The stability, equilibrium, device recovery and PPB were calculated using the following equations (Lindup & Orme, 1981):

(5) $$\%\;\text{Remaining at}\;5\;h=\frac{{\text{Concentration}}_{4\;h}}{{\text{Concentration}}_{0\;h}}\times 100$$

(6) $$\%\;\text{Equilibrium}=\frac{{\text{Concentration}}_{\text{receiver cell}}}{{\text{Concentration}}_{\text{donor cell}}}\times 100$$

(7) $$\%\;\text{Device Recovery}=\frac{{\text{Concentration}}_{\text{receiver cell}}+{\text{Concentration}}_{\text{donor cell}}}{{\text{Concentration}}_{0\;h}}\;\;\times 100$$

(8) $$\%\;\text{Bound}=\frac{{\text{Concentration}}_{\text{donor cell}}-{\text{Conentration}}_{\text{receiver cell}}}{{\text{Concentration}}_{\text{donor cell}}}\times 100$$

#### 2.8.4 ∣ *In vitro* metabolic stability in liver S9 fraction and microsomes

Metabolic stability was assessed using mouse, rat and human liver microsomes, and mouse liver S9 fractions (XenoTech, LLC, Lenexa, KS, USA) were used for the *in vitro* assessment of phase I and II metabolism. Incubation with S9 fractions was performed in triplicate, at 2 μm SF2523 concentration as described in our previous report (Aldhafiri et al., 2020; Bala et al., 2020). The reaction was started by adding 2 μl of SF2523 at a final concentration of 2 μm. Serial samples (50 μl) were collected at selected time intervals and quenched with 300 μl of methanol spiked with 10 μl of IS (0.5 μg/ml). All of the samples were vortexed and centrifuged at 13,000**g** for 15 min, and the supernatant was collected and transferred to an autosampler vial and injected (2 μl) onto the LC–MS/MS system. Testosterone 7-hydroxycoumarin and diclofenac were used as positive controls to ensure that S9, microsomes and the incubation conditions were appropriate to conduct metabolism studies.

The S9 fraction and microsomal stability were expressed as the percentage of drug remaining at each time. The *in vitro* metabolic elimination rate constant was determined from the first-order plot of a natural logarithm of the area ratio vs. time. The slope of the linear regression equation was used to determine the elimination rate constant *k* (min^−1^).

#### 2.8.5 ∣ Metabolite identification

For metabolite identification, mass shift analyses were performed using the same LC–MS/MS system. The mobile phase and column as described in section 2.2 were also used for metabolite identification using a 35 min gradient time profile via Q1 scan mode in a range of 200–1,000 Da as described in our previous report (Aldhafiri et al., 2020; Bala et al., 2020). The parent drug MS/MS spectrum was used as a template model for structure identification and assuming that the fragmentation pattern of the parent drug from the production spectrum was used to deduce the structures of the metabolites (Francis et al., 1999). The data acquisition for the metabolite identification study was conducted using Labsolution software (Shimadzu Scientific Instruments, Columbia, MD, USA, version 5.8).

## 3 ∣ RESULTS

### 3.1 ∣ Chromatographic and mass spectrometric conditions optimization

**FIGURE 1:**
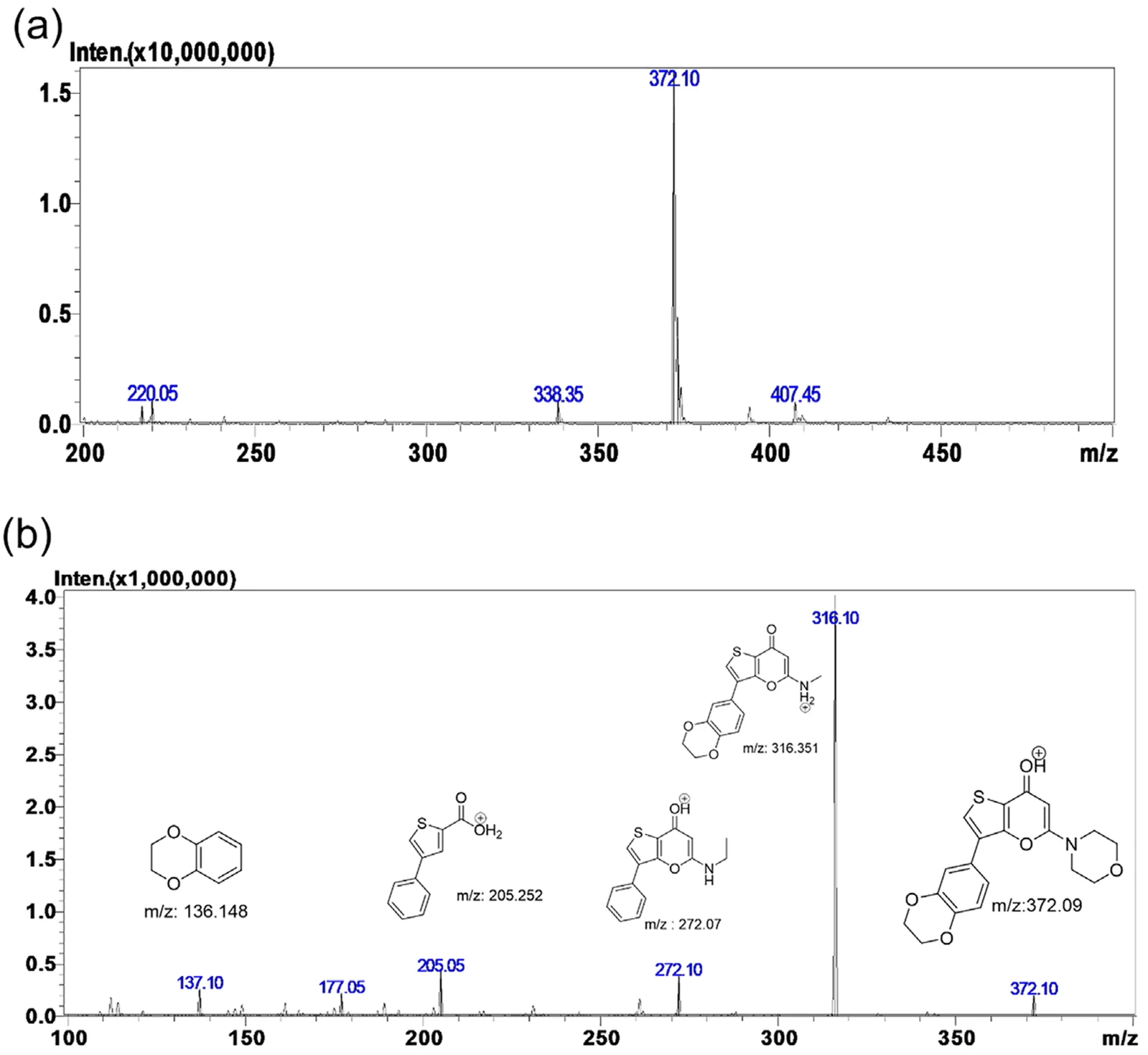
Mass spectra of SF2523. (a) MS spectra of SF2523 in positive ESI mode show a prominent parent peak at *m/z* 372.10 and (b) MS/MS product ion spectra of SF2523 in positive ESI mode show prominent fragments peak at *m/z* 316.1, 272.1 and 137.1.

To optimize mass spectrometric conditions for quantitation of SF2523 and IS, electrospray ionization (ESI) and atmospheric pressure chemical ionization conditions were tested. The MS/MS signal intensities of SF2523 and IS were found to be the highest using ESI in the positive MRM mode (data not shown). MS/MS conditions were optimized using 1 μg/ml of SF2523. The major product ions from protonated molecular ions of SF2523 (*m/z* 372.10) and IS (*m/z* 433.05) were observed at *m/z* 316.05 for SF2523 and *m/z* 341.15for IS, respectively (Figure 1a,b). The three most sensitive MRM pair ions were observed at *m/z* 372.10 > 316.05, 372.10 > 205.05 and 372.10 > 137.15. The resulting MRM ions were used as the primary ions for quantitation of SF2523 and IS: 372.10 > 316.05 and 433.05 > 341.15, respectively.

**FIGURE 2:**
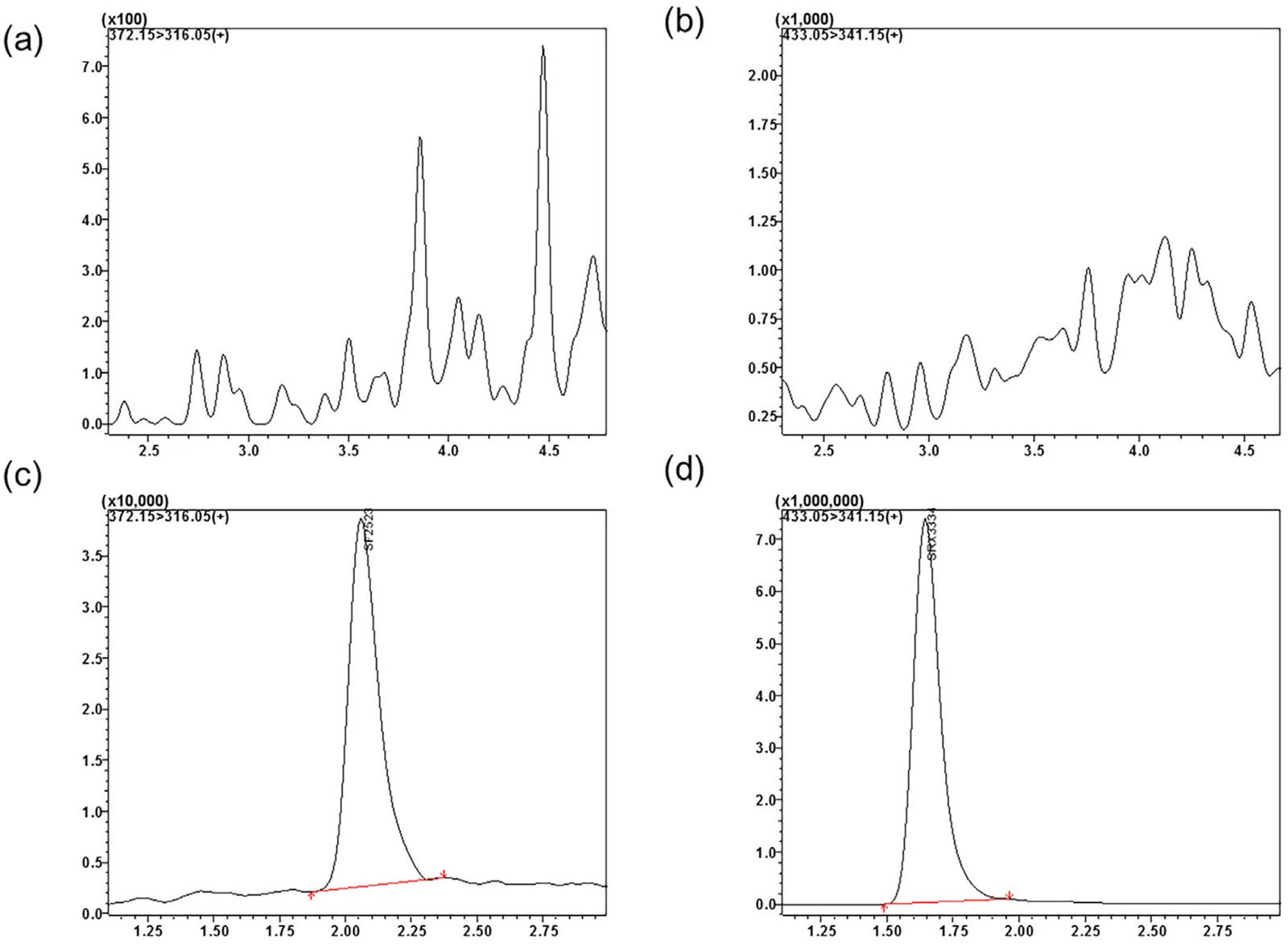
Representative MRM ion chromatograms of (a) blank mouse plasma using the conditions for SF2523 detection, (b) blank mouse plasma using the conditions for IS detection, (c) plasma spiked with SF2523 at LQC (retention time 2.0 min, 0.3 ng/ml) and (d) plasma spiked with IS (retention time 1.7 min, 1,000 ng/ml).

Various chromatographic conditions were investigated to attain high resolution, good peak shape and short retention time, such as mobile phase (acetonitrile, methanol and water), additives (acetic acid, ammonium acetate, formic acid and formate), gradient parameters and the analytical column (C_8_, C_18_ and C_18_ pentafluorophenyl [PFP]). Finally, the ACE Excel C_18_ column (see Materials and Methods section) with the following mobile phase conditions yielded an optimal balance of retention times between SF2523 (2.0 min) and the IS (1.70 min). No analytical interference was observed at the retention times of the analyte and the IS, and no visible peaks from endogenous compounds were observed in the blank and spiked plasma samples (Figure 2).

The SPE method was used for plasma sample cleanup for LC–MS/MS analysis of SF2523. Before, the final SPE protocol, we optimized several extraction methods including protein precipitation, liquid–liquid extraction (LLE) and solid-phase extraction (SPE). The SPE method using Bond Elut C_18_ columns was selected as the extraction method. Conversely, the SPE method provided an efficient sample clean-up in comparison with LLE (data not shown). Mean recoveries were >80% utilizing the final SPE method for both analyte and IS.

### 3.2 ∣ Assay validation

Typical LC–MS/MS chromatograms of blank plasma samples and blank plasma samples spiked with SF2523 and IS are presented in Figure 2. No analytical interference or visible peak from endogenous compounds was observed at the retention times of SF2523 (2.0 min) and IS (1.7 min).

The linearity of the LC–MS/MS method was determined by the CCs analyzed on three independent days. All results were fitted using a 1/*x*^2^ weighted linear regression. High linearity was observed for SF2523 in the range of 0.1–500 ng/ml (coefficient of determination ≥ 0.998). Samples spiked with 0.1 ng/ml were found to have the lowest concentration with RSD < 20%, and thus the LLOQ was defined as 0.1 ng/ml. In the carryover sample, no significant peaks (≥20% LLOQ) were detected at the retention times of SF2523 and IS (data not shown). Thus, it was concluded that carryover was not an issue with the current method.

The intra- and inter-batch precision and accuracy of SF2523 were assessed at LLOQ and the three QC levels were found to be within the acceptable limits as per the FDA guidelines (Table 2). The RSD of precision values ranged from 1.63 to 13.95%, indicating acceptable assay precision. The accuracy of the quantitative analysis of the compounds varied from −10.03 to 11.56%, within the acceptance limits at all concentration levels. The experimental results showed that the method was reproducible, reliable and suitable for the determination of SF2523 in mouse plasma.

### 3.3 ∣ Recovery and matrix effect

The recoveries for SF2523 and IS from spiked plasma samples were calculated using LQC, MQC and HQC concentrations. The percentage mean recoveries for SF2523 in plasma at three QC levels were 88.32 ± 9.72, 92.63 ± 10.69 and 81.41 ± 6.72%, respectively (Table 3). The mean recovery of IS was 92.62 ± 3.93%. Furthermore, the ME and IS-normalized ME for SF2523 were measured at concentrations of three QC levels. At the three QC levels, the MF values of SF2523 ranged from 98.9 to 107.65 (<±15%), indicating that the plasma matrix components did not affect the determination of analytes in mouse plasma.

### 3.4 ∣ Stability

The plasma stability results of SF2523 are summarized in Table 4. SF2523 was stable in mouse plasma when kept at room temperature for 6 h, at −80°C for 60 days’ long storage and after three freeze–thaw cycles. The processed plasma samples were stable in an auto-sampler at 15°C for 24 h. The results showed that the plasma samples were stable in the process of preparation and storage, and the appropriateness of the current method.

### 3.5 ∣ *In vitro* studies

#### 3.5.1 ∣ Gastrointestinal stability of SF2523

SF2523 was found to be stable at gastrointestinal pH, 1.2 and 6.8, as well as at physiological pH 7.4 using phosphate buffer with >90% of the parent remaining after 1 h (SGF) and 2 h of incubation under all of the tested pH conditions (1.2, 6.8 and 7.4).

#### 3.5.2 ∣ The blood-to-plasma ratio

The blood-to-plasma ratio (B/P) ratio was determined for SF2523 at concentrations of 0.1 and 1 μg/ml, and was were 0.88 and 0.90, respectively (Table 5).

#### 3.5.3 ∣ Plasma protein binding

SF2523 was highly bound to mouse plasma proteins (>98%) at both tested concentrations (Table 6). Moreover, non-specific binding (>90% recovery) was not observed with the membrane or equilibrium device and SF2523 was stable in mouse plasma with 5 h of incubation (Table 6). Positive control drugs propranolol (high protein binding) and atenolol (low protein binding) were within the acceptable limits reported in the literature (Barber et al., 1978).

#### 3.5.4 ∣ *In vitro* metabolic stability in microsomes

**FIGURE 3:**
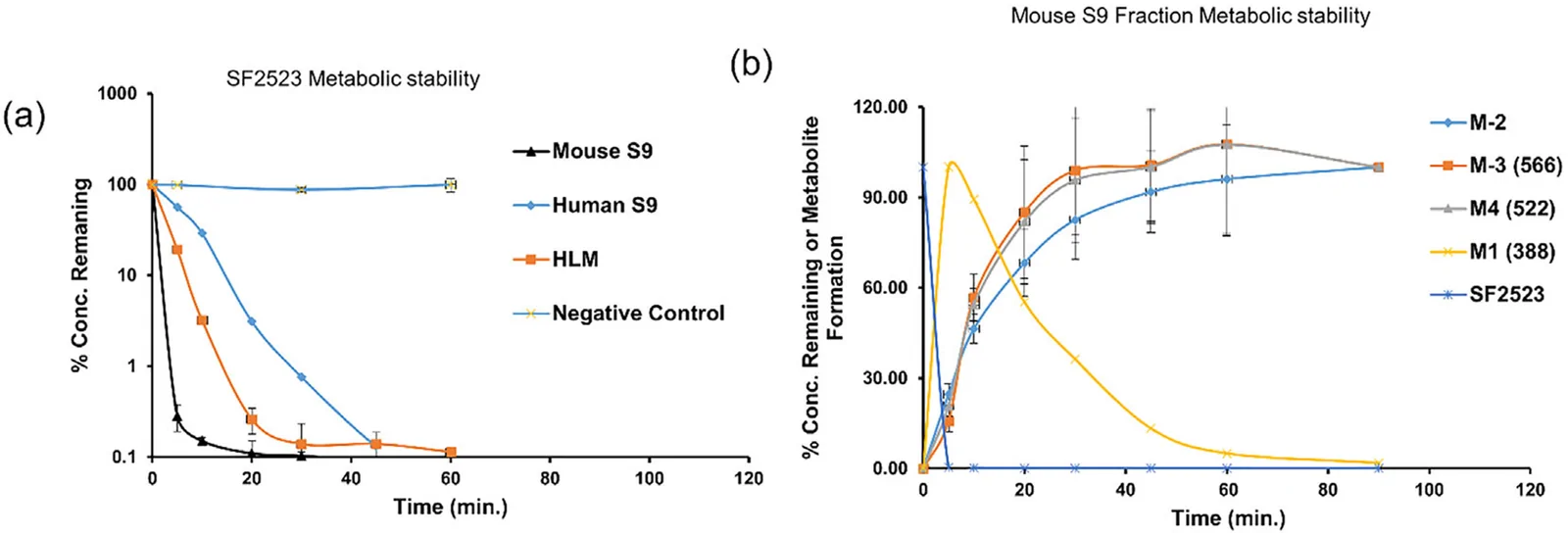
Time-dependent metabolic depletion (percentage turnover or amount of drug remaining vs. incubation time) of SF2523 in microsomes and mouse S9 fraction. (a) Metabolic elimination profiles (percentage turnover or amount remaining vs. incubation time) for reaction with SF2523 in human liver microsomes, mouse and human S9 and negative control. (b) Mouse S9 metabolic stability of SF2523 in the presence of NADPH and metabolite formation. Data are shown as mean ± SD (n = 3).

The *in vitro* metabolic stability of SF2523 was investigated using mouse and human S9 and liver microsomes. The result of the metabolic stability study was expressed as the percentage parent remaining at different time points relative to the parent at 0 min (100% parent) and metabolite formation (Figure 3a,b). The *in vitro t*_1/2_ of SF2523 was <2 min in mouse and human S9 and liver microsomes. Positive controls utilizing testosterone, 7-hydroxycoumarin and diclofenac were within acceptable in-house limits.

#### 3.5.5 ∣ Metabolite identification

**FIGURE 4:**
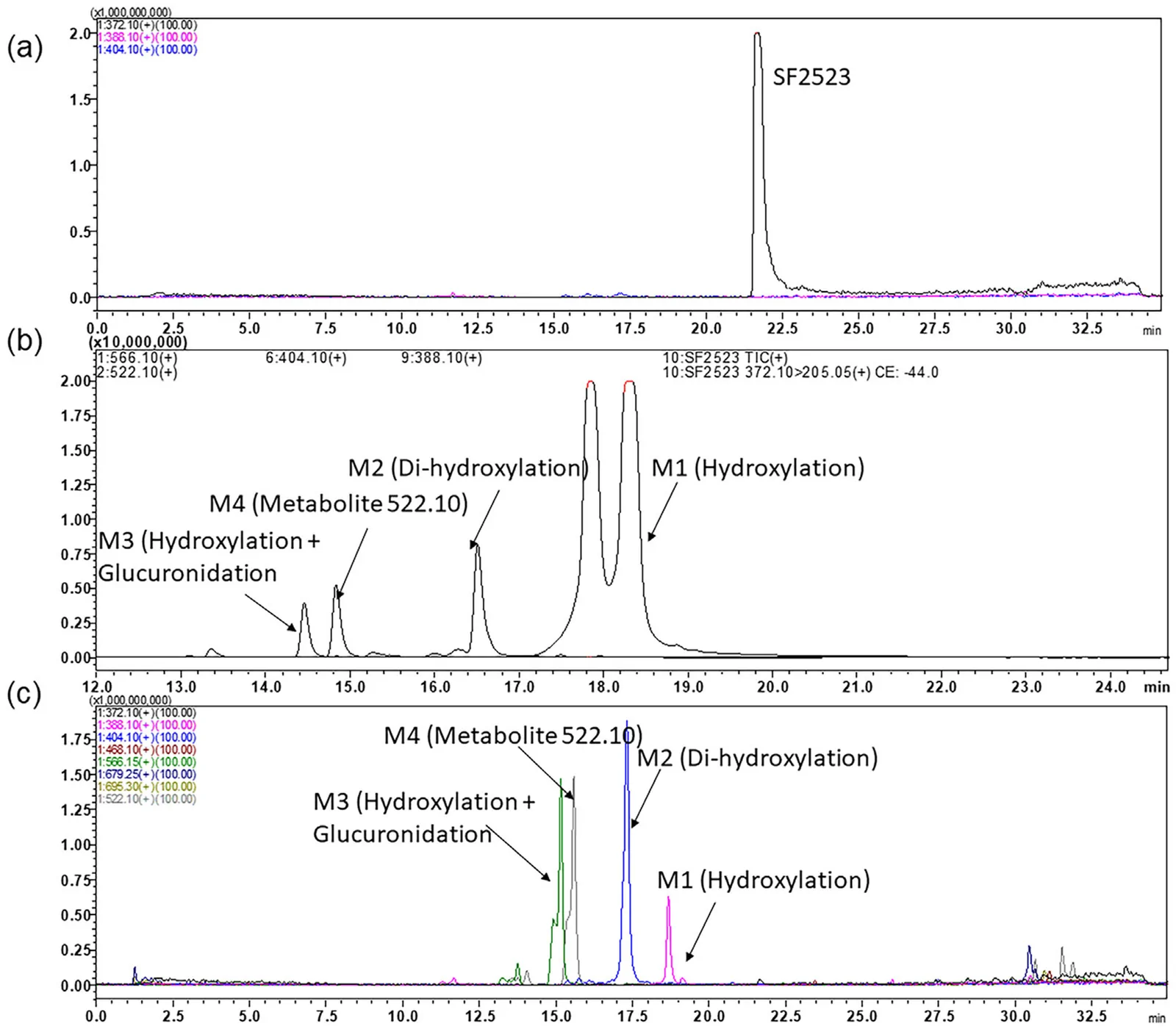
Representative overlay chromatogram of SF2523 and its metabolites under 35 min gradient chromatography after (a) 0 min of incubation, (b) 5 min of incubation and (c) 90 min of incubation.

SF2523 samples from mouse S9 fraction run in a 35 min gradient method using LC–MS/MS in ESI mode (Figure 4). The retention time was noted for every peak along with the precursor ion peak [M + H]^+^, and structural information was collected by fragmentation of all precursor ions. The exact mass of each predicted metabolite was used to generate the MS/MS fragmentation of each metabolite via a manual product ion scan.

The protonated molecular ion [M + H]^+^ of SF2523 was detected at 22.40 min (retention time) with an *m/z* of 372.10. Seven SF2523 metabolites (Table 7) were identified along with their putative structure via plausible fragmentation (Figure S1) based on their LC/MS data, precursor ion and retention times.

Two major metabolites (M1) were identified just after 5 min of incubation in the mouse S9 fraction. The M1 metabolite (*m/z* 388) is suggested to be a mono-hydroxylated product with 16 Da higher molecular weight than the parent compound (*m/z* 372) (Figure S2). The fragment of M1 at *m/z* 288 is also comparable with the parent ion, indicating mono-hydroxylation on the morpholine ring system. However, the presence of two LC peaks for M1 metabolite at 5 min shows an equal possibility of hydroxy attachment at different positions of the morpholine ring system. The M2 metabolite (*m/z* 404) has 32 Da higher molecular weight than the parent (*m/z* 372), indicating di-hydroxylation (Figure S3). Similarly, for M1, diagnostic fragments (*m/z* 386, 345, 288) also confirm the position of dihydroxylation on the morpholine ring.

**FIGURE 5:**
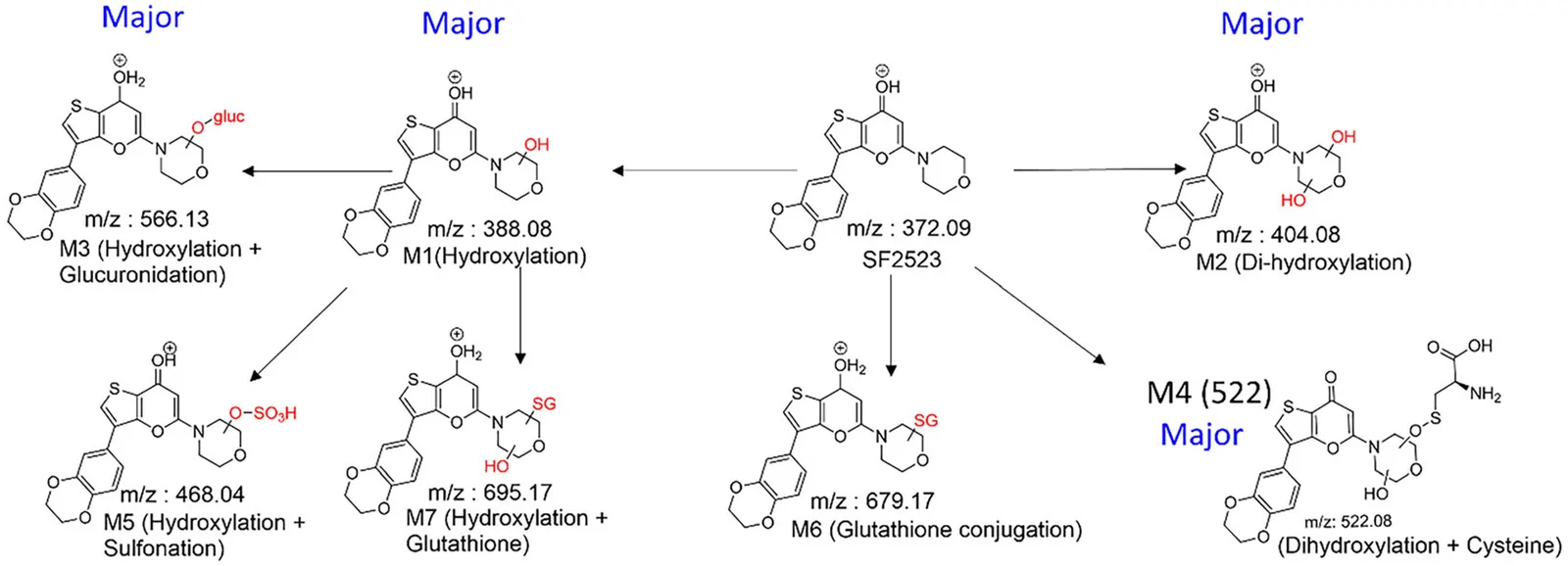
Structures of proposed putative SF2523 metabolites.

Along with M1 and M2, five other metabolites (M3, M4, M5, M6 and M7) were identified (Figure 5). Metabolite M3 (*m/z* 566) suggests O-glucuronidation with a 194 Da increase in molecular weight compared with the parent structure (Figure S4). The fragment ion of M3 at *m/z* 388 validates mono-hydroxylation on the morpholine ring system. Metabolite M4 (*m/z* 522) suggests di-hydroxylation along with cysteine conjugation with a 150 Da increase in the parent peak (Figure S5). The structure of metabolite M5 indicates O-sulfonation (*m/z* 468) over the mono-hydroxylated metabolite (*m/z* 388) (Figure S6). Two minor metabolites (M6 and M7) were identified as glutathione conjugates on the morpholine ring (M6, *m/z* 679) and mono-hydroxylation along with glutathione conjugation (M7, *m/z* 695) with mass shifts of 307 and 323 Da compared with the parent compound (*m/z* 372), respectively.

## 4 ∣ DISCUSSION

We successfully developed and validated a sensitive, selective and rapid LC–MS/MS method for the quantitation of SF2523 in mouse plasma. The MS/MS response was linear in the range 0.1–500 ng/ml with a correlation coefficient (*r*^2^ > 998) for all calibration curves in 100 μl of plasma. The method was validated according to the FDA guidelines showing accuracy and precision within the acceptance limits set forth in FDA (2018).

Auto-optimization using the ESI source in positive mode was used to detect the precursor and MRM for SF2523 and IS, resulting in lower background noise and acceptable sensitivity for the quantitation of SF2523. The analyte and IS were chromatographically resolved by reverse-phase chromatography (C_18_ column), using a mobile phase constituting methanol and 0.1% formic acid at a flow rate of 0.25 ml/min. The retention time was short (2.0 min), with a good peak shape and high analyte sensitivity. Plasma sample extraction and cleanup were achieved using SPE. The SPE method was developed while taking into consideration the drug’s physical–chemical properties and compatibility with the chosen LC method. The extraction method was reproducible with consistently high extraction recovery averaging >80% for SF2523 and >90% for IS and negligible matrix effect.

The SF2523 was found to be stable (>90% of parent drug remaining) under a variety of storage conditions. Along with the stability of the compound in the mouse plasma, it was stable in GI fluids such as SIF, SGF and PBS at 37°C. The stability results showed that the plasma samples as well as GI fluids were stable during preparation and storage, and the current method is appropriate. SF2523 was found to have high plasma proteins binding (>95%) to mouse plasma. The free drug concentration in systemic circulation is considered to be a pharmacologically and toxicologically active fraction (Lindup & Orme, 1981). The B/P ratio of SF2523 was <1 (0.85–0.98). The B/P ratio indicates that the SF2523 does not partition in the blood. Therefore, we can use plasma/serum as the biological matrix to accurately detect the SF2523 concentration in the systemic circulation. At a B/p ratio of >1 (usually as a consequence of the drug distributing into the erythrocytes), the plasma clearance significantly overestimates blood clearance and could exceed hepatic blood flow (Saha et al., 2017). Additionally, the metabolic stability of SF2523 was evaluated in mouse and human S9 fraction and liver microsomes. In an *in vitro* mouse, S9 metabolic investigations exhibited rapid metabolic degradation with seven metabolites being detected, with hydroxylation being the major metabolic pathway. Hydroxylated major metabolites (M1; showing two positional isomeric peaks) were detected within 5 min of incubation in the mouse S9 fraction. The M1 metabolite at 5 min showed an equal possibility of hydroxy attachment at different positions of the morpholine ring system. These hydroxylated metabolites appear to behave as a parent and further linearly degrade into submetabolites (Figure 4b). The results of these *in vitro* drug-metabolism studies provide the necessary impetus for further structural modification of this pharmacophore to improve stability and pave the way for the rational development of its analogs. The acute and chronic toxicities associated with SF2523 or one of its resulting metabolites are still unclear so preliminary toxicology studies in mice are crucial. Future studies will investigate individual CYP or UGT involvement, fractionation, and structure identification to check the pharmacological and toxicity behavior of metabolites.

## 5 ∣ CONCLUSION

A sensitive, efficient and reproducible method for the quantitation of the novel dual PI3K/BRD4 inhibitor SF2523 in mouse plasma was developed and validated. Furthermore, the assay was sensitive (LLOQ 0.1 ng/ml utilizing 100 μl of the sample), linear over a 3-fold logarithmic range and displayed low intra-run and inter-run variability. SF2523 was rapidly metabolized by the mouse S9 fraction and microsomes, with seven metabolites being detected, with hydroxylation being the major metabolic pathway. The combination of these factors results in a rapid and reliable method of quantitation of SF2523 and permits the translation of this method as a high-throughput means for large-scale murine studies. Future efforts will focus on the disposition and activity of metabolites of SF2523, and the in vivo efficacy of SF2523 may be a result of the compound itself, metabolites, or a combination of both. This work provided useful data and a reliable method to support further pharmacological or toxicological studies as well as clinical research.

## Supplementary Material

Supplementary Figures

Additional supporting information can be found online in the Supporting Information section at the end of this article.

## Figures and Tables

**TABLE 1:** Summary of optimized MS/MS parameters: precursor ions, fragment ions, the voltage potential (Q1), collision energy (CE), the voltage potential (Q3) and retention time for the analyte and internal standard.

Analytes	MRM transition *m/z* (Q1 > Q3)	Q1 (V)	CE (V)	Q3 (V)	Retention time (min)
SF2523	372.10 > 316.05	−30	−35	−24	3.5
	372.10 > 205.05	−21	−44	−15	
	372.10 > 137.15	−30	−51	−28	
SRX3334 (IS)	433.05 > 341.15	−19	−39	−17	3.1

Abbreviation: MRM, Multiple reaction monitoring.

**TABLE 2:** Intra- and inter-day precision (RSD) and accuracy (bias) for SF2523 in mouse plasma.

Level	Nominal concentration (ng/ml)	Intra-day		Inter-day	
		Bias (%)	RSD (%)	Bias (%)	RSD (%)
LLOQ	0.1	−3.28	1.63	−6.33	6.14
LQC	0.3	−10.03	13.95	−10.56	8.41
MQC	200	7.55	3.41	12.87	7.19
HQC	375	9.45	9.95	11.56	6.80

Abbreviations: HQC, high quality control; LLOQ, Lower limit of quantitation; LQC, low quality control; MQC, mediun quality control.

**TABLE 3:** Assessment of the recovery and matrix effect of SF2523 in mouse plasma, (mean ± SD, *n* = 3).

Nominal concentration (ng/ml)	Percentage extraction recoveries (mean ± SD, *n* = 3)	Percentage matrix effect (mean ± SD, *n* = 3)
LQC (0.1 ng/ml)	88.32 ± 9.72	104.43 ± 6.35
MQC (200 ng/ml)	92.63 ± 10.69	98.9 ± 3.35
HQC (375 ng/ml)	81.41 ± 6.72	102.59 ± 1.68
Internal standard (IS): (25 ng/ml)	92.62 ± 3.93	107.65 ± 6.20

**TABLE 4:** Mean stability recoveries of SF2523 under different storage conditions in mouse plasma.

Storage conditions	Nominal concentration (ng/ml)	SF2523 (mean ± SD, *n* = 3)	
		Measured mean concentration (ng/ml)	Accuracy (%)
Bench-top stability for 8 h at (21°C, 6 h)	0.3	0.29 ± 0.01	95.75 ± 4.03
	200	234.08 ± 9.34	107.05 ± 4.5
	375	416.76 ± 0.71	111.15 ± 0.21
Autosampler stability (4°C, 24 h)	0.3	0.33 ± 0.03	104.85 ± 5.16
	200	201.74 ± 3.59	100.85 ± 1.77
	375	385.45 ± 4.3	102.8 ± 1.13
Three freeze-thaw cycles (−80°C to room temperature)	0.3	0.28 ± 0.04	92.9 ± 12.3
	200	202.7 ± 10.21	101.35 ± 5.0
	375	426.45 ± 0.68	113.7 ± 0.14
Long-term stability (60 days, −80°C)	0.3	0.31 ± 0.01	102.75 ± 2.62
	200	205.81 ± 22.41	102.9 ± 11.17
	375	364.67 ± 1.36	97.25 ± 0.35

**TABLE 5:** The values of SF2523 blood-to-plasma ratio (B/P) in mouse blood.

Time point (min)	At 0.1 μg/ml B/P	At 1 μg/ml B/P
0	0.85	0.85
30	0.98	0.93
60	0.83	0.93

Abbreviation: B/P, blood-to-plasma ratio.

**TABLE 6:** Plasma protein binding of SF2523 in mouse plasma (*n* = 3, mean ± SD).

Parameters	SF2523	
	Mean	SD
Percentage remaining at 5 h (stability)	99.06	9.44
Percentage device recovery	98.58	7.33
Percentage plasma protein bound (at 1 μg/ml)	95.82	0.36
Percentage plasma protein bound (at 10 μg/ml)	96.73	0.96

**TABLE 7:** Proposed metabolites and respective biotransformation, chemical structure, precursor ion, and retention times.

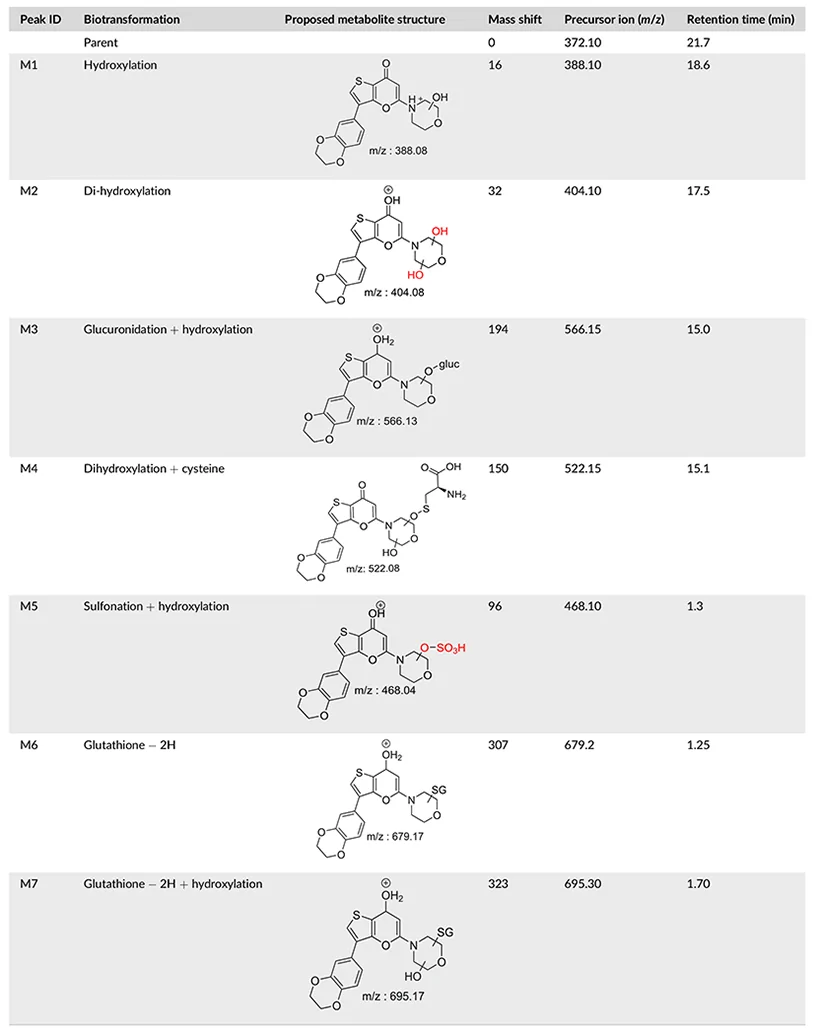

## Abbreviations

APCI: atmospheric pressure chemical ionization
B/P ratio: blood-to-plasma
BET: bromodomain and extraterminal domain
CCs: calibration curve
CE: collision energy
CL int: intrinsic clearance
CSs: calibration standards
DUIS: dual ion source
EICs: extracted ion chromatograms
FA: formic acid
FA: formic acid
HIV-1: human Immunodeficiency virus type-1
HLM: human liver microsome
HQC: high quality control
IS: internal standard
LC-MS/MS: Liquid Chromatography with tandem mass spectrometry
LLE: liquid-liquid extraction
LLOQ: lower limit of quantification
LQC: low quality control
ME: matrix effect
MQC: middle quality control
MRM: multiple reaction monitoring
PI3K: phosphoinositide 3-kinases
PPB: plasma protein binding
QC: quality control
RED: rapid equilibrium dialysis
S/N: signal/noise
SGF: simulated gastric fluid
SPE: solid phase extraction
t_1/2_: half-life
TP: thienopyranone
UHPLC: ultra-high performance liquid chromatography

## References

[R1] Aldhafiri WN, Chhonker YS, Zhang Y, Coulter DW, McGuire TR, Li R, & Murry DJ. (2020). Assessment of tissue distribution and metabolism of MP1, a novel pyrrolomycin, in mice using a validated LC-MS/MS method. Molecules, 25(24), 5898. https://www.mdpi.com/1420-3049/25/24/5898. 10.3390/molecules25245898 33322110 PMC7764159

[R2] Andrews FH, Singh AR, Joshi S, Smith CA, Morales GA, Garlich JR, Durden DL, & Kutateladze TG. (2017). Dual-activity PI3K-BRD4 inhibitor for the orthogonal inhibition of MYC to block tumor growth and metastasis. Proceedings of the National Academy of Sciences, 114(7), E1072–E1080. 10.1073/pnas.1613091114 PMC5320964 28137841

[R3] Bala V, Chhonker YS, Sleightholm RL, Crawford AJ, Hollingsworth MA, & Murry DJ. (2020). A rapid and sensitive bioanalytical LC–MS/MS method for the quantitation of a novel CDK5 inhibitor 20–223 (CP668863) in plasma: Application to in vitro metabolism and plasma protein-binding studies. Biomedical Chromatography, 34(8), e4859. 10.1002/bmc.4859 32307720 PMC10664148

[R4] Bandukwala HS, Gagnon J, Togher S, Greenbaum JA, Lamperti ED, Parr NJ, Molesworth AM, Smithers N, Lee K, & Witherington J. (2012). Selective inhibition of CD4+ T-cell cytokine production and autoimmunity by BET protein and c-Myc inhibitors. Proceedings of the National Academy of Sciences, 109(36), 14532–14537. 10.1073/pnas.1212264109 PMC3437860 22912406

[R5] Barber H, Hawksworth G, Kitteringham N, Petersen J, Petrie J, & Swann J. (1978). Protein binding of atenolol and propranolol to human serum albumin and in human plasma [proceedings]. British Journal of Clinical Pharmacology, 6(5), 446P–447P. 10.1111/j.1365-2125.1978.tb04617.x 728297

[R6] Boehm D, Calvanese V, Dar RD, Xing S, Schroeder S, Martins L, Aull K, Li P-C, Planelles V, & Bradner JE. (2013). BET bromodomain-targeting compounds reactivate HIV from latency via a Tat-independent mechanism. Cell Cycle, 12(3), 452–462. 10.4161/cc.23309 23255218 PMC3587446

[R7] Campbell GR, Bruckman RS, Herns SD, Joshi S, Durden DL, & Spector SA. (2018). Induction of autophagy by PI3K/MTOR and PI3K/MTOR/BRD4 inhibitors suppresses HIV-1 replication. Journal of Biological Chemistry, 293(16), 5808–5820. 10.1074/jbc.RA118.002353 29475942 PMC5912471

[R8] Carlino L, & Rastelli G. (2016). Dual kinase-bromodomain inhibitors in anticancer drug discovery: A structural and pharmacological perspective. Journal of Medicinal Chemistry, 59(20), 9305–9320. 10.1021/acs.jmedchem.6b00438 27559828

[R9] Dai X, Gan W, Li X, Wang S, Zhang W, Huang L, Liu S, Zhong Q, Guo J, & Zhang J. (2017). Prostate cancer–associated SPOP mutations confer resistance to BET inhibitors through stabilization of BRD4. Nature Medicine, 23(9), 1063–1071. 10.1038/nm.4378 PMC5625299 28805820

[R10] Delmore JE, Issa GC, Lemieux ME, Rahl PB, Shi J, Jacobs HM, Kastritis E, Gilpatrick T, Paranal RM, & Qi J. (2011). BET bromodomain inhibition as a therapeutic strategy to target c-Myc. Cell, 146(6), 904–917. 10.1016/j.cell.2011.08.017 21889194 PMC3187920

[R11] FDA. (2018, May). Drug Administration Centre for Drug Evaluation and Research (FDA). Guidance for industry-bioanalytical method validation. Center for Drug Evaluation and Research, US Department for Health and Human Services.

[R12] Filippakopoulos P, & Knapp S. (2014). Targeting bromodomains: Epigenetic readers of lysine acetylation. Nature Reviews Drug Discovery, 13(5), 337–356. 10.1038/nrd4286 24751816

[R13] Francis B, Yves Le Blanc JC, Coutu M, Ramier I, Moreau JP, & Brown NK. (1999). Metabolite profiling study of propranolol in rat using LC/MS/MS analysis. Biomedical Chromatography, 13(5), 363–369. 10.1002/(SICI)1099-0801(199908)13:5<363::AID-BMC894>3.0.CO;2-G 10425029

[R14] Gallenkamp D, Gelato KA, Haendler B, & Weinmann H. (2014). Bromodomains and their pharmacological inhibitors. ChemMedChem, 9(3), 438–464. 10.1002/cmdc.201300434 24497428

[R15] Janku F, Yap TA, & Meric-Bernstam F. (2018). Targeting the PI3K pathway in cancer: Are we making headway? Nature Reviews Clinical Oncology, 15(5), 273–291. 10.1038/nrclinonc.2018.28 29508857

[R16] Joshi S, Singh AR, Liu KX, Pham TV, Zulcic M, Skola D, Chun HB, Glass CK, Morales GA, Garlich JR, & Durden DL. (2019). SF2523: Dual PI3K/BRD4 inhibitor blocks tumor immunosuppression and promotes adaptive immune responses in cancer. Molecular Cancer Therapeutics, 18(6), 1036–1044. 10.1158/1535-7163.MCT-18-1206 31018997 PMC6893301

[R17] Laplante M, & Sabatini DM. (2012). mTOR signaling in growth control and disease. Cell, 149(2), 274–293. 10.1016/j.cell.2012.03.017 22500797 PMC3331679

[R18] Lindup W, & Orme M. (1981). Clinical pharmacology: Plasma protein binding of drugs. British Medical Journal (Clinical Research Ed.), 282(6259), 212–214. 10.1136/bmj.282.6259.212 6779954 PMC1503970

[R19] Matuszewski BK, Constanzer ML, & Chavez-Eng CM. (1998). Matrix effect in quantitative LC/MS/MS analyses of biological fluids: A method for determination of finasteride in human plasma at picogram per milliliter concentrations. Analytical Chemistry, 70, 882–889. 10.1021/ac971078+ 9511465

[R20] Morales GA, Garlich JR, Su J, Peng X, Newblom J, Weber K, & Durden DL. (2013). Synthesis and cancer stem cell-based activity of substituted 5-morpholino-7H-thieno[3,2-b]pyran-7-ones designed as next generation PI3K inhibitors. Journal of Medicinal Chemistry, 56(5), 1922–1939. 10.1021/jm301522m 23410005

[R21] Rathert P, Roth M, Neumann T, Muerdter F, Roe J-S, Muhar M, Deswal S, Cerny-Reiterer S, Peter B, & Jude J. (2015). Transcriptional plasticity promotes primary and acquired resistance to BET inhibition. Nature, 525(7570), 543–547. 10.1038/nature14898 26367798 PMC4921058

[R22] Saha A, Kumar A, Gurule S, Khuroo A, & Srivastava P. (2017). Role of RBC partitioning and whole blood to plasma ratio in bioanalysis: A case study with valacyclovir and acyclovir. Mass Spectrom. Purif. Tech, 3(2), 119. 10.4172/2469-9861.1000119

[R23] Saxton RA, & Sabatini DM. (2017). mTOR signaling in growth, metabolism, and disease. Cell, 168(6), 960–976. 10.1016/j.cell.2017.02.004 28283069 PMC5394987

[R24] Shimobayashi M, & Hall MN. (2014). Making new contacts: The mTOR network in metabolism and signalling crosstalk. Nature Reviews. Molecular Cell Biology, 15(3), 155–162. 10.1038/nrm3757 24556838

[R25] Thorpe LM, Yuzugullu H, & Zhao JJ. (2015). PI3K in cancer: Divergent roles of isoforms, modes of activation and therapeutic targeting. Nature Reviews. Cancer, 15(1), 7–24. 10.1038/nrc3860 PMC4384662 25533673

[R26] Vann KR, Pal D, Morales GA, Burgoyne AM, Durden DL, & Kutateladze TG. (2020). Design of thienopyranone-based BET inhibitors that bind multiple synthetic lethality targets. Scientific Reports, 10(1), 12027. 10.1038/s41598-020-68964-6 32694708 PMC7374098

[R27] Vidler LR, Brown N, Knapp S, & Hoelder S. (2012). Druggability analysis and structural classification of bromodomain acetyl-lysine binding sites. Journal of Medicinal Chemistry, 55(17), 7346–7359. 10.1021/jm300346w 22788793 PMC3441041

[R28] Vlahos CJ, Matter WF, Hui KY, & Brown RF. (1994). A specific inhibitor of phosphatidylinositol 3-kinase, 2-(4-morpholinyl)-8-phenyl-4H-1-benzopyran-4-one (LY294002). The Journal of Biological Chemistry, 269(7), 5241–5248. 10.1016/S0021-9258(17)37680-9 8106507

[R29] Wang J, Yadav V, Smart AL, Tajiri S, & Basit AW. (2015). Toward oral delivery of biopharmaceuticals: An assessment of the gastrointestinal stability of 17 peptide drugs. Molecular Pharmaceutics, 12(3), 966–973. 10.1021/mp500809f 25612507

[R30] Wymann MP, & Schneiter R. (2008). Lipid signalling in disease. Nature Reviews. Molecular Cell Biology, 9(2), 162–176. 10.1038/nrm2335 18216772

[R31] Zhang P, Wang D, Zhao Y, Ren S, Gao K, Ye Z, Wang S, Pan C-W, Zhu Y, & Yan Y. (2017). Intrinsic BET inhibitor resistance in SPOP-mutated prostate cancer is mediated by BET protein stabilization and AKT–mTORC1 activation. Nature Medicine, 23(9), 1055–1062. 10.1038/nm.4379 PMC5653288 28805822

