# Supplementary material for: LC–MS/MS method for the quantitation of a dual PI3K/BRD4 inhibitor SF2523 in mouse plasma: Application to plasma protein binding and metabolism studies
Source: Biomed Chromatogr. Author manuscript; Available in PMC 2026 Aug 20. (PMC13492205; doi:10.1002/bmc.5643)
Supplement: Supplementary file 1 [file NIHMS2200870-supplement-Supplementary_Figures.docx]

**LC-MS/MS method for the quantitation of a dual PI3K/BRD4 inhibitor SF2523 in mouse plasma: application to plasma protein binding and metabolism Studies**

Veenu Bala^1,#^, Yashpal S. Chhonker^1,#,∆^, Guillermo A. Morales^2^, Krishnaiah Maddeboina^3,6^, Dhananjaya Pal^3^, Donald L. Durden^3,4,6^, Daryl J. Murry^1,5,∆^

^1^ Clinical Pharmacology Laboratory, Department of Pharmacy Practice and Science, University of Nebraska Medical Center, Omaha, NE 68198, USA; [Veenu2bala@gmail.com](mailto:Veenu2bala@gmail.com) (V.B.) [y.chhonker@unmc.edu](mailto:y.chhonker@unmc.edu) (Y.S.C.)

^2^ SignalRx Pharmaceuticals, Inc.; Cumming, GA 30041, USA, Email; [morales@signalrx.com](mailto:morales@signalrx.com) (G.A.M.)

^3^ Molecular Targeted Therapeutics Laboratory, Atrium Health-Levine Cancer Institute; Charlotte, NC, 28204, USA; Krishnaiah.Maddeboina@atriumhealth.org (K.M.), [dhananjaya.pal@atriumhealth.org](mailto:dhananjaya.pal@atriumhealth.org) (D.P.)

^4^ Division of Pediatric Hematology-Oncology, Department of Pediatrics, Moores Cancer Center, UC San Diego School of Medicine; La Jolla, CA 92130, USA.[Donald.Durden@atriumhealth.org](mailto:Donald.Durden@atriumhealth.org) (D.L.D.)

^5^ Fred and Pamela Buffett Cancer Center, University of Nebraska Medical Center, Omaha, NE; [dj.murry@unmc.edu](mailto:dj.murry@unmc.edu) (D.J.M.)

^6^Atrium Health Wake Forest Baptist Comprehensive Cancer Center, Winston-Salem, NC 27157, USA

*^#^ Authors contributed equally to this manuscript*

^∆^ Corresponding authors:

Daryl J. Murry, Pharm. D. (E-mail: [dj.murry@unmc.edu](mailto:dj.murry@unmc.edu))

Yashpal Singh Chhonker, Ph.D. (E-mail: [y.chhonker@unmc.edu](mailto:y.chhonker@unmc.edu))

Clinical Pharmacology Laboratory

Department of Pharmacy Practice and Science, College of Pharmacy

University of Nebraska Medical Center

Omaha, NE 68198-6025

Phone: 402-559-3790 (office), 402-559-2430 (lab)

**Figure S1.** Product ion spectra of **SF2523** and proposed fragmentation pattern of precursor ion (M+H).

**Figure S2.** Product ion spectra of proposed metabolite (hydroxylation, M1) and diagnostic MS fragment ions via plausible fragmentation pattern.

**Figure S3.** Product ion spectra of proposed metabolite (di-hydroxylation, M2) and diagnostic MS fragment ions via plausible fragmentation pattern.

**Figure S4.** Product ion spectra of proposed metabolite (glucuronidation + hydroxylation, M3) and diagnostic MS fragment ions via plausible fragmentation pattern.

**Figure S5.** Extracted ion LC chromatogram and product ion spectra of proposed metabolite (di-hydroxylation **+** Cysteine, M4) and diagnostic MS fragment ions via plausible fragmentation pattern.

**Figure S6.** Product ion spectra of proposed metabolite (sulfonation + hydroxylation, M5) and diagnostic MS fragment ions via plausible fragmentation pattern.

**Figure S7.** Product ion spectra of proposed metabolite (glutathione-2H, M6) and diagnostic MS fragment ions via plausible fragmentation pattern.

**Figure S8.** Product ion spectra of proposed metabolite (glutathione-2H + hydroxylation, M7) and diagnostic MS fragment ions via plausible fragmentation pattern.
